# A child with resistant Kawasaki disease successfully treated with anakinra: a case report

**DOI:** 10.1186/s12887-017-0852-6

**Published:** 2017-04-08

**Authors:** J. Sánchez-Manubens, A. Gelman, N. Franch, S. Teodoro, J. R. Palacios, N. Rudi, J. Rivera, J. Antón

**Affiliations:** 1 Pediatric Rheumatology Unit, Pediatrics Department, Hospital Parc Taulí Sabadell, Barcelona, Spain; 2 Pediatric Rheumatology Unit, Pediatrics Department, Hospital Sant Joan de Déu Esplugues, Barcelona, Spain; 3 Pediatrics Department, Hospital Parc Taulí Sabadell, Barcelona, Spain; 4 Pediatric Cardiology Unit, Pediatrics Department, Hospital Parc Taulí Sabadell, Barcelona, Spain; 5 Pharmacy Department, Hospital Parc Taulí Sabadell, Barcelona, Spain

**Keywords:** Kawasaki disease, IVIG resistance, Anakinra, IL1 blockade, Case report

## Abstract

**Background:**

Kawasaki disease (KD) is an acute self-limited systemic vasculitis of unknown etiology. Intravenous immunoglobulin (IVIG) is an effective treatment and decreases the risk of cardiac complications to less than 5%. In spite of its effectiveness, some children do not respond to this therapy and still develop coronary aneurysms (CAA). The optimal treatment for IVIG non-responsive patients remains controversial although corticoids have been suggested to be an effective treatment in some patients. For those patients still resistant to IVIG and corticoids, interleukin-1 receptor antagonists (IL-1RA) such anakinra could be an alternative.

**Case presentation:**

We present a 3 year-old Caucasian patient with KD without cardiac complications but with important resistance to treatment. After becoming resistant to IVIG and corticoids, anakinra proved to be an effective treatment.

**Conclusions:**

To our knowledge, this is the first report of the utility of IL-1RA in refractory KD without coronary impairment. The patient fulfilled the classical criteria for KD and, after becoming resistant to first and second line treatments, anakinra proved to be an effective treatment. Further studies are required to determine if this is an effective treatment option for other cases of resistant Kawasaki disease.

## Background

KD is an acute self-limited systemic vasculitis of unknown etiology presenting predominantly in toddlers and children under 5 years old. Diagnosis is based on clinical criteria including fever, exanthema, conjunctivitis, changes in hands and feet, erythema of oral mucosa and lips and cervical lymphadenopathy. Prognosis depends on the extent of cardiac involvement; CAA develop in 20-25% of untreated patients and these may lead to myocardial infarction and sudden death if proper treatment with IVIG is not administered within the first 10 days of illness [1, 2]. Early treatment with IVIG decreases the risk of cardiac complications to less than 5%. In spite of its effectiveness, some children do not respond to this therapy and still develop CAA. Although the optimal treatment for IVIG non-responsive patients remains controversial, adding steroids to the 2nd IVIG dose has proven to be effective to reduce the incidence of CAA and improve the prognosis of resistant KD when administered in patients fulfilling IVIG resistance criteria in the Kobayashi scoring system [3, 4]. For those who present a lack of response to this 2nd step of treatment, other approaches have been described, such as infliximab [5], plasma exchange [6] or cyclosporine [7]. In the last years some reports have suggested the role of IL-1RA in the treatment of severe or resistant cases of KD [8, 9]. We present a case of a IVIG and steroids resistant KD successfully treated with IL-1RA (anakinra).

## Case presentation

We present a previously healthy 3 year-old Caucasian girl, who was admitted with persistent fever for 5 days, generalized rash, non-purulent conjunctivitis, labial and lingual erythema and swollen feet. On admission, the girl had significant irritability. Blood tests showed normal hemoglobin, white blood cells and platelets, high C-reactive protein (CRP - 14 mg/dL) and high transaminases (AST 168 U/L, ALT 86 U/L). Fulfilling the KD classical criteria, KD was diagnosed and treatment with IVIG (2 g/kg) and aspirin (100 mg/kg) was initiated. Echocardiography two days after admission showed mild mitral and tricuspideal regurgitation but no CAA. Electrocardiogram was normal.

Despite the initial IVIG treatment, fever, rash, conjunctivitis and labial and lingual erythema remained, and 2 additional IVIG doses and 2 metilprednisolone (30 mg/kg) pulses were administered in the subsequent days. Eight days after admission, fever disappeared and analytical features normalized.

**Fig. 1:**
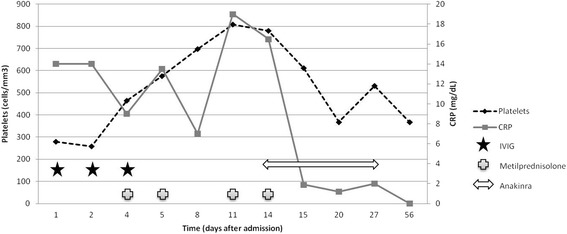
Time course of C reactive protein (CRP), platelets and treatment

Maintenance treatment with oral prednisone (0.5 mg/kg/day) was initiated but, on day 11 after admission, fever recurred with important irritability, exanthema and hand and feet desquamation. An important increase on CRP (16.5 mg/dL), ESR (126 mm/h) and platelets (808000) was registered and there was a decrease in hemoglobin (7.5 g/dL) (Fig. 1). Echocardiography did not show changes. Another 2 metilprednisolone pulses were administered without response. On day 14 after admission (19 days since onset), due to conventional treatment failure and having ruled out, together with the patient’s parents, the use of other intravenous treatments such as infliximab, IL-1RA was initiated (anakinra - 2 mg/kg subcutaneous once a day for 14 days). Fever and irritability disappeared within hours and CRP, ESR, platelets and hemoglobin became normal in the subsequent blood tests (24 h and 5 days after treatment initiation).

Six days after anakinra was initiated the patient was discharged. Treatment was maintained for 2 weeks combined with aspirin. No relapses appeared after anakinra discontinuation and subsequent blood tests and echocardiographies at weeks 2, 8 and 16 after discharge were normal (no changes on coronary arteries sizes have been recorded over time and z-scores have maintained between 1 and 1.5 SDs). The patient did not experience any side effects or complications during or after the use of anakinra.

## Discussion

In the last years, some studies have suggested an important role for interleukin 1 (IL-1) in the pathogenesis of KD [10]. Genome-Wide Association Studies (GWAS) have also found functional SNPs in *ITPKC* and *CASP3* genes that are associated with an increased risk of unresponsiveness to IVIG therapy [11]. Lee et al. suggested, using a mouse model of KD, that IL-1 receptor-deficient mice were protected from induced coronary lesions. Furthermore, daily injections of the IL-1RA prevented induced coronary lesions in normal mice [12].

Another study developed in a cohort of Taiwanese children with KD, found a significant increase in IVIG resistance risk in those patients with the IL-1B −511 TT and IL-1B −31 CC genotypes and the diplotype TC/TC in the IL-1 family of genes [13]. These results suggest an important genetic association between IL-1 and failure of initial IVIG therapy and support the previous findings that IL-1 secretion is associated with IVIG treatment in KD [14, 15].

Anakinra is a recombinant antagonist of the IL-1 receptor used successfully to treat systemic onset juvenile idiopathic arthritis. Regarding the clinical application of the exposed findings on the role of IL-1 in the pathogenesis of KD, two clinical trials are being held in Europe and the USA and two case reports on KD patients treated with anti-Il-1 have been reported [8, 9]. Both papers describe severe cases of KD, one of them complicated with a macrophage activation syndrome (MAS), that were resistant to multiple IVIG and prednisolone pulses and improved drastically after the administration of anakinra with analytical normalization and complete reversal of the echocardiogram changes. We present a case report in which the patient fulfilled the classical criteria for KD but had no cardiac or severe complications and, after becoming resistant to first and second line treatments, IL-1R blockade proved to be an effective treatment. Further studies are required and are being held to determine if this is an effective treatment option for all cases of resistant Kawasaki disease.

## Conclusion

To our knowledge, this is the first report on the utility of IL-1RA in refractory KD without coronary impairment or MAS. The patient fulfilled the classical criteria for KD and, after becoming resistant to first and second line treatments, IL-1R blockade proved to be an effective treatment without side effects or complications. Further studies are required to determine if this is an effective treatment option for other cases of resistant Kawasaki disease.

## Abbreviations

ALT: Alanine aminotransferase
AST: Aspartate aminotransferase
CAA: Coronary aneurysms
CRP: C-reactive protein
ESR: Erythrocyte sedimentation rate
IL-1RA: Interleukin-1 receptor antagonists
IVIG: Intravenous immunoglobulin
KD: Kawasaki disease
MAS: Macrophage activation syndrome
